# A pan-cancer analysis targeting the oncogenic role of ATP-binding cassette transporter A1 in human tumors

**DOI:** 10.3389/fonc.2025.1513992

**Published:** 2025-04-14

**Authors:** Huidan Wu, Shinong Gu, Shuyuan Xie, Xuanwen Li, Bingye Wang, Yuxuan Liu, Qing Huang

**Affiliations:** ^1^ College of Environment and Public Health, Xiamen Huaxia University, Xiamen, Fujian, China; ^2^ Department of Clinical Nutrition, Tianjin Beichen Traditional Chinese Medicine Hospital, Tian Jin, China

**Keywords:** ABCA1, pan-cancer, survival, prognosis, bioinformatics

## Abstract

**Objectives:**

ABCA1 is involved in the development and progression of a wide range of malignant tumors, so to further clarify the role of ABCA1 expression therein, and to search for new breakthroughs in the treatment of tumors and cancers, we launched a thorough pan-cancer analysis of ABCA1.

**Methods:**

Based on the manipulation of TCGA (The Cancer Genome Atlas), GEO (Gene Expression Omnibus), Human Protein Atlas (HPA) datasets and various bioinformatics tools, The oncogenic role of ABCA1 in 33 tumor types was explored from six aspects: gene expression, prognosis, variation, immunohistochemistry, correlation of tumor-associated fibroblastic infiltration, and enriched analysis of related genes. The potential value of ABCA1 was also mined, such as: ABCA1 may be a potential marker of tumor metastasis, play a role in cancer resistance, and its expression may inhibit the spread of tumor cells.

**Results:**

Based on the analysis results, we found that ABCA1 is expressed elevated and mutated in tumor samples of 33 cancer types compared with matched normal tissues, and these mutations may be related to the mechanism of cancer, metastatic ability, and prognosis, etc. Meanwhile, we also investigated the correlation between ABCA1 expression and tumor-associated fibroblast infiltration, and described its association with corresponding miRNAs, which can provide scientific basis for clinical diagnosis.

**Conclusions:**

Our study thoroughly illustrates the impact of ABCA1’s systemic presence across various forms of cancer. Given its specificity to tumors, ABCA1 holds promise as a biomarker for cancer diagnosis, monitoring for recurrence, and predicting outcomes. Consequently, our research could significantly bolster the case for utilizing ABCA1 in the therapeutic approach to cancer.

## 1 Introduction

Cholesterol is an important component of cell membranes and a precursor to certain hormones, and its balance is essential for the normal physiology of all cells, in addition to the synthesis and transport of cholesterol, which is a key factor in the development of cancer. There is evidence that hypercholesterolemia is associated with an increased risk of colorectal, prostate, and other cancers, and a high-cholesterol diet has been shown to promote cancer development. An important factor affecting intracellular cholesterol levels is protein transport (1). ATP-binding cassette transporter A1 (ABCA1), a major transporter that regulates intracellular cholesterol, is a protein involved in cholesterol balance and is widely expressed as a transmembrane protein in many tissues (2). It can transfer intracellular free cholesterol and phospholipids to apolipoprotein A-I (apoA-I), generate newborn high-density lipoprotein (nHDL) particles (3), and exert its anti-inflammatory effects by modifying membrane lipid rafts and directly activating signalling pathways (4). It uses ATP as its energy source, and its gene, which is one of the key members of the ATP binding box transporter superfamily, is highly polymorphic (5). Moreover, the significant differences in mRNA and protein expression patterns suggest that ABCA1 expression may be posttranscriptionally regulated.

The structure and mechanism of action of ABCA1 clearly influence its function in normal cells, and conversely, it also reflects its interventional effect on pathological cells. According to existing studies, these results suggest that HDL by an ABCA1-dependent mechanism can mediate signal transduction, leading to increased proliferation and migration of prostate cancer cells (6). NNMT promotes high metastasis in triple-negative breast cancer by enhancing membrane fluidity via the PP2A/MEK/ERK/c-Jun/ABCA1 pathway (7). *In vitro*, gastric adenocarcinoma cells with ABCA1 knockdown exhibited significantly inhibited proliferation and invasion properties (8). These studies suggest that ABCA1 may influence cancer cell proliferation, metastasis, and invasion to some extent. In addition, ABCA1 may be important for the treatment of cancer. For example, ABCA1 plays a key role in drug resistance and prognosis (9). Ovarian cancer patients with high RASSF1C methylation (which plays an oncogenic role in cancer cells) and low ABCA1 expression have shorter survival (10). ABCA1-mediated EMT promotes thyroid carcinoma malignancy through the ERK/Fra-1/ZEB1 pathway (11). ABCA1 has been associated with the development of acquired chemotherapy resistance and the prediction of adverse outcomes in ovarian cancer patients, indicating the potential role of ABCA1 in cancer treatment (12). Therefore, in general, ABCA1 is associated with cancer proliferation, invasion, metastasis, and changes in cancer treatment. However, there is still no reliable evidence confirming the pan-cancer relationship between ABCA1 and various tumour types on the basis of clinical big data indicators. In view of the complexity of tumorigenesis, pan-cancer expression analysis is very important.

To address this situation, our study combined the cancer genomic dataset TCGA and the high-throughput gene expression database GEO to conduct a pan-cancer analysis of the ABCA1 protein, and since many previous studies have been published, a series of factors, such as gene expression, survival status, gene variation, immune infiltration and related cell pathways, were fully considered. Therefore, we wrote this review to explore the mechanism of action of ABCA1 in the pathogenesis and prognosis of different types of cancer, as well as its potential value in clinical diagnosis and treatment.

## 2 Materials and methods

### 2.1 Gene expression analysis

We entered *ABCA1* into the “Gene_DE” module of the TIMER2 [Tumor Immune Estimation Resource (version 2)] site (http://timer.cistrome.org/) and observed *ABCA1* expression between different tumours or specific tumour subtypes in the TCGA project. For some tumours with no or highly limited normal tissues [e.g., TCGA-GBM (glioblastoma multiforme), TCGA-LAML (eosinophilic leukaemia), etc.], we used the “Expression Analysis Box Plot” module (http://gepia2.cancer-pku.cn/#analysis) of the GEPIA2 (Gene Expression Profiling Interactive Analysis version 2) web server to compare these tumour tissues with the GTEx normal tissue (genotype-tissue expression) database box plots to reflect differences in expression, setting a *P* value cut-off = 0.01, a log2FC (fold change) cut-off = 1, and “matching TCGA normal and GTEx data”. In addition, we obtained violin maps of *ABCA1* in different pathological stages (stages I–IV of all TCGA tumours) via the “Pathological Stage Plots” module of GEPIA2. The expression data converted from log2 [TPM (transcripts per million) +1] were applied to box plots or violin plots. Moreover, the UCSC Xena Browser (https://xenabrowser.net/) was used to perform progression-free survival (PFS) analysis of *ABCA1* using TCGA Pan-Cancer datasets (version: 2018–09–13). The expression threshold was set at 50% for high *ABCA1* expression and low *ABCA1* expression. Displaying the genomic information and visualizing the relevant data of the *ABCA1* gene is conducive to our comprehensive analysis.

### 2.2 Survival prognosis analysis

To clarify the genetic prognostic value of *ABCA1*, we used the “Survival Map” module of the GEPIA2 web server to obtain significance mapping data for overall survival (OS) and disease-free survival (DFS) associated with *ABCA1* in all TCGA tumours. Critical high (50%) and critical low (50%) values were used as expression thresholds to differentiate between high- and low-expression cohorts (13). Hypothesis testing was performed using the log-rank test to classify high- and low-expression tumours into high- and low-expression groups, and survival maps were obtained from the “Survival Analysis” module of GEPIA2. Hazard ratios and log-rank *P* values were also calculated via the purified Spearman rank correlation test and other statistical tests.

### 2.3 Genetic alteration analysis

After logging into cBioPortal Web (https://www.cbioportal.org/), we selected “TCGA Pan-Cancer Atlas Study” in the “Quick Select” section and entered “*ABCA1*” to query the genetic alteration characteristics of *ABCA1* and obtain more information about the characteristics of *ABCA1* gene changes. We then selected the ‘Cancer Type Summary’ module, from which we observed the data on the frequency of changes, mutation types, and CNA (copy number changes) of all TCGA tumours. With the “Mutations” module, the mutation site information of *ABCA1* can be displayed in the protein structure schematic or three-dimensional (3D) structure picture. We also utilized the “Comparison” module to obtain data on the differences in overall survival, disease-specific survival, disease-free survival, and progression-free survival of TCGA cancer patients with and without *ABCA1* gene alterations. Moreover, we also generated Kaplan–Meier plots with log-rank *P* values to further clarify the genetic variation in *ABCA1*.

### 2.4 Immunohistochemistry staining

To assess the differences in ABCA1 expression at the protein level more clearly, we entered the HPA (human protein mapping) (http://www.proteinatlas.org/) database, input “ABCA1” and then clicked the “Tissue” or “Pathology” module, after which the corresponding data can be downloaded. We downloaded IHC images of ABCA1 protein expression in normal tissues and 6 tumour tissues and systematically analysed them. IHC can be used to detect the presence of target antigens in cells or tissues. These tumours included LUAD (lung adenocarcinoma), BRCA (breast invasive carcinoma), OV (ovarian plasma cystic adenocarcinoma), LIHC (hepatocellular carcinoma), TGCT (testicular germ cell tumour), and THCA (thyroid cancer) tissues. We then used the “Expression Analysis Box Plot” module of the GEPIA2 web server to select each tumour type and obtained a block diagram of expression differences between these 6 tumour tissues and normal tissues..

### 2.5 Immune infiltration analysis

We used the “immune-gene” module of the TIMER2 web server to explore the relevance of *ABCA1* to the infiltration of all tumour-associated fibroblasts in TCGA. We selected immune cells as CD8^+^ T cells and cancer-associated fibroblasts. The TIMER, CIBERSORT, CIBERSORT-ABS, QUANTISEQ, XCELL, MCPCOUNTER, and EPIC algorithms were applied to estimate immune infiltration (14). P values and partial correlation (cor) values were derived using purity-adjusted Spearman rank correlation tests. The data were visualized as heatmaps and scatter plots (15).

### 2.6 ABCA1-related gene enrichment analysis

To investigate the functional associations of ABCA1, we utilized the STRING database (https://string-db.org/) to explore protein interactions. Specifically, we queried for the protein “ABCA1” in *Homo sapiens* with the following parameters: a minimum interaction score of “low confidence” (0.150), edge evidence from experimental data, a maximum of 50 interactors, and interactions sourced from experimental evidence. This process enabled us to identify ABCA1-binding proteins based on available experimental data. We then successfully screened 50 existing experimentally proven ABCA1-binding proteins. We then imported the diagram obtained from the STRING website into Cytoscape software to modify it and obtain **Figure 6D**.

Using the TCGA dataset encompassing various tumour and normal tissue samples, we employed the “Similar Gene Detection” module of GEPIA2 to identify the top 100 genes most closely associated with *ABCA1*. Subsequent Pearson correlation analysis was performed using the “Correlation Analysis” module in GEPIA2, with the results depicted in dot plots based on log^2^-transformed TPM values. The plots included *P* values and correlation coefficients (R). Additionally, heatmap data representing the expression profiles of selected genes were generated using the “Gene Corr” module of TIMER2, including partial correlations and *P* values derived from the purity-adjusted Spearman rank correlation test (16).

After the above two sets of data were obtained, we combined and filtered them and used the online gene function annotation analysis tool Metascape (http://metascape.org/gp/) to submit 150 genes successively to the main interface of Metascape (17). The species *H. sapiens* and Express Analysis were selected to download the Excel data, and then the following four data of the first 15 channels in Excel were used: pathway (description column of the table), enrichment (result of the formula in the InTerm_InList column of the table), *P* value, and count (first number of the formula in the InTerm_InList column of the table). Then, the “enrichment dot bubble” module of Wei Sheng Xin (http://www.bioinformatics.com.cn/) was used to input the data we sorted. The X-axis is the Rich factor, the colour is -log10(*P* value), and the dot size is the count. The results were then sorted according to the *P* value, and a bubble map was obtained to clarify and carry out visual analysis of the enrichment path. Two-tailed *P* < 0.05 was considered statistically significant.

In addition, to display the pan-cancer genes and pathways related to the *ABCA1* gene, we selected the five pathways with the most genes by selecting the “Linear dendrogram” graphical module via the Raw Graphs 2.0 (https://app.rawgraphs.io) website. A Sankey diagram of *ABCA1* is generated.

### 2.7 *CircAB1* gene upstream miRNA prediction

To describe the link between the *ABCA1* gene and the corresponding microRNAs, we adopted the starBase database (https://starbase.sysu.edu.cn/), selected the “micrornas - Target” module, clicked on the “micrornas - mRNA” option, inputted our target gene “*ABCA1*”, downloaded the table, and screened it through Excel software. Representative miRNA targets were selected according to the number of predicted databases to explain the corresponding relationship (prediction by three or more databases was considered to represent correlation). To improve visualization, the miRNA-ABCA1 regulatory network was established using Cytoscape software.

## 3 Results

### 3.1 *ABCA1* expression is upregulated in multiple tumours

**Figure 1:**
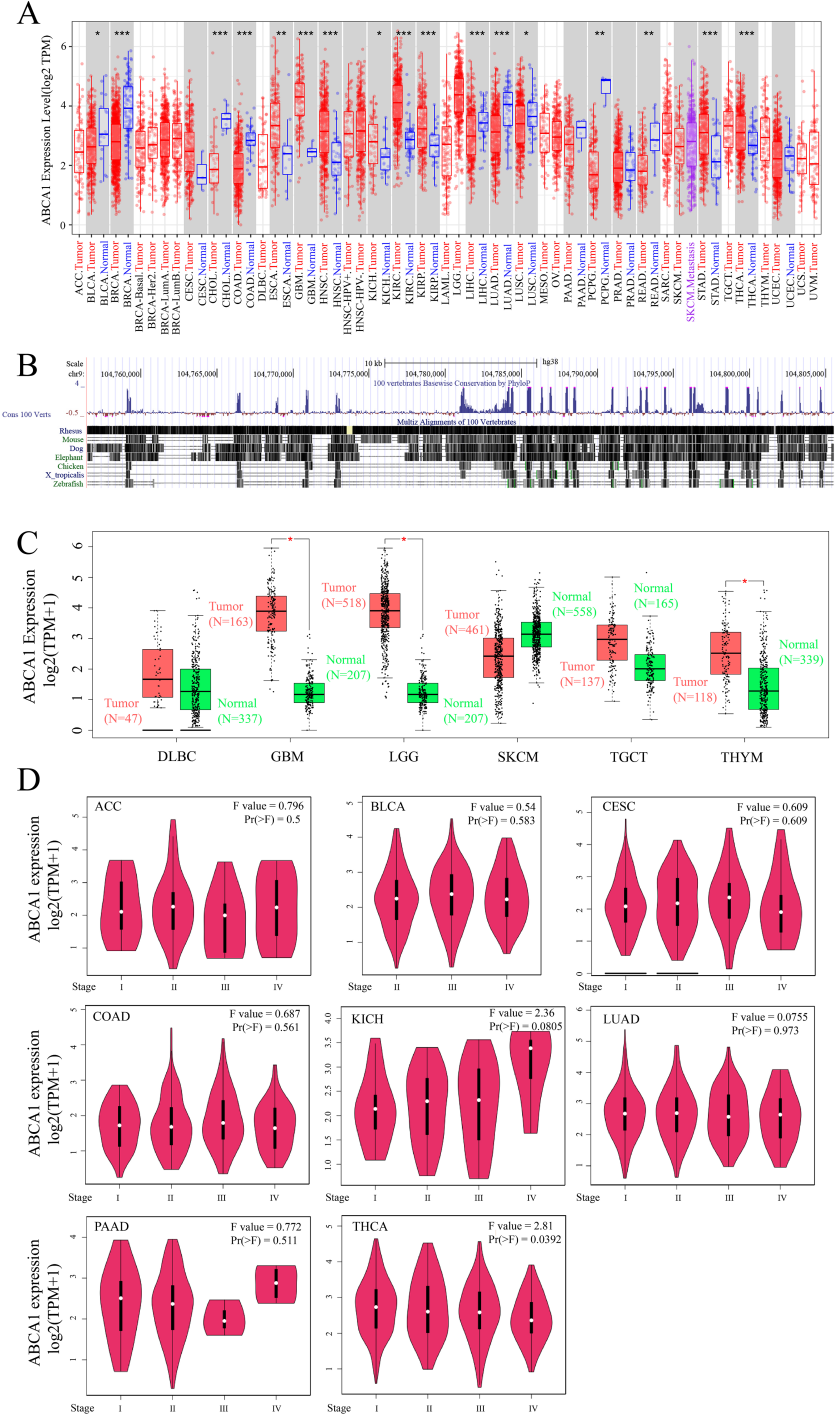
Expression level of *ABCA1* gene in different tumors and pathological stages. **(A)** The expression status of the *ABCA1* gene in different cancers or specific cancer subtypes was analyzed through TIMER2. *P < 0.05; **P < 0.01; ***P < 0.001. **(B)** *ABCA1* gene conservation analysis among vertebrates was visualized using the UCSC genome browser. **(C)** For the type of DLBC (Tumor N = 47; Normal N = 337), GBM (Tumor N = 163; Normal N = 207), LGG (Tumor N = 518; Normal N = 207), SKCM (Tumor N = 461; Normal N = 558), TGCT (Tumor N = 137; Normal N = 165), and THYM (Tumor N = 118; Normal N = 339) in the TCGA project, the corresponding normal tissues of the GTEx database were included as controls. The box plot data were supplied. *P < 0.05. **(D)** Based on the TCGA data, the expression levels of the *ABCA1* gene were analyzed by the main pathological stages (stage I, stage II, stage III, and stage IV) of ACC, BLCA, CESC, COAD, KICH, LUAD, PAAD, and THCA. Log2 (TPM+1) was applied for log-scale.

We integrated tumour samples and normal samples from the TCGA database, analysed the expression levels of *ABCA1* in each TCGA cancer type, and determined the expression characteristics of *ABCA1* mRNA using the TIMER2 method. As shown in **Figure 1A**, the expression levels of *ABCA1* in various tumour tissues, such as GBM (glioblastoma multiforme) (*P* < 0.001), HNSC (head and neck squamous cell carcinoma) (*P* < 0.001), KIRC (clear cell carcinoma of kidney) (*P* < 0.001), KIRP (papillary cell carcinoma of kidney) (*P* < 0.001), STAD (gastric adenocarcinoma) (*P* < 0.001), THCA (thyroid carcinoma) (*P* < 0.001), ESCA (oesophageal carcinoma) (*P* < 0.01), and KICH (phaeochromocytoma of kidney) (*P* < 0.05), were greater than those in normal tissues, whereas the expression levels of ABCA1 in BRCA (breast invasive carcinoma) (*P* < 0.001), CHOL (cholangiocarcinoma) (*P* < 0.001), COAD (colon adenocarcinoma) (*P* < 0.001), LIHC (liver hepatocellular carcinoma) (*P* < 0.001), LUAD (lung adenocarcinoma) (*P* < 0.001), PCPG (pheochromocytoma and paraganglioma) (*P* < 0.01), READ (rectal adenocarcinoma) (*P* < 0.01), BLCA (urothelial carcinoma of the bladder) (*P* < 0.05), and LUSC (squamous cell carcinoma of the lung) (*P* < 0.05) tumour tissues were lower than in normal tissues.

Since these results, we utilized the UCSC Genome Browser to analyse and perform multiple comparisons of genome sequences from different species. As shown in **Figure 1B**, *ABCA1* is relatively conserved in vertebrates.

Using normal tissues in the GTEx dataset as controls, we investigated the differences in the expression of ABCA1 between normal tissues and tumour tissues. As shown in **Figure 1C**, the expression levels of ABCA1 in SKCM (skin melanoma) (*P* > 0.05) tumour tissues were clearly lower than that in normal tissues, whereas the expression levels of ABCA1 in GBM (glioblastoma multiforme) (*P* < 0.05), LGG (low-grade glioma) (*P* < 0.05), THYM (thymic carcinoma) (*P* < 0.05), DLBC (diffuse large B-cell lymphoma) (*P* > 0.05), and TGCT (tenosynovial giant cell tumour) (*P* > 0.05) tumour tissues were higher than that in normal tissues.

As shown in **Figure 1D**, ACC (adenoid cystic carcinoma), BLCA (bladder uroepithelial carcinoma), CESC (cervical squamous carcinoma), COAD (colorectal carcinoma), KICH (renal pheochromocytoma), LUAD (lung adenocarcinoma), PAAD (primary intrahepatic cholangiocarcinoma), and THCA (thyroid carcinoma) are cancers whose pathological staging correlates with the expression of ABCA1. We used the “pathological stage map” module of HEPIA2 to observe the correlation between ABCA1 expression and the pathological stage of cancers, such as ACC (adenoid cystic carcinoma) and BLCA (bladder urothelial carcinoma). The correlation was directly reflected in the form of a violin map (**Figure 1D**, only THCA had a *P* value of < 0.05, KICH had a *P* value of < 0.1, and the rest had *P* values of > 0.5).

### 3.2 *ABCA1* expression is related to the prognosis of various tumours

**Figure 2:**
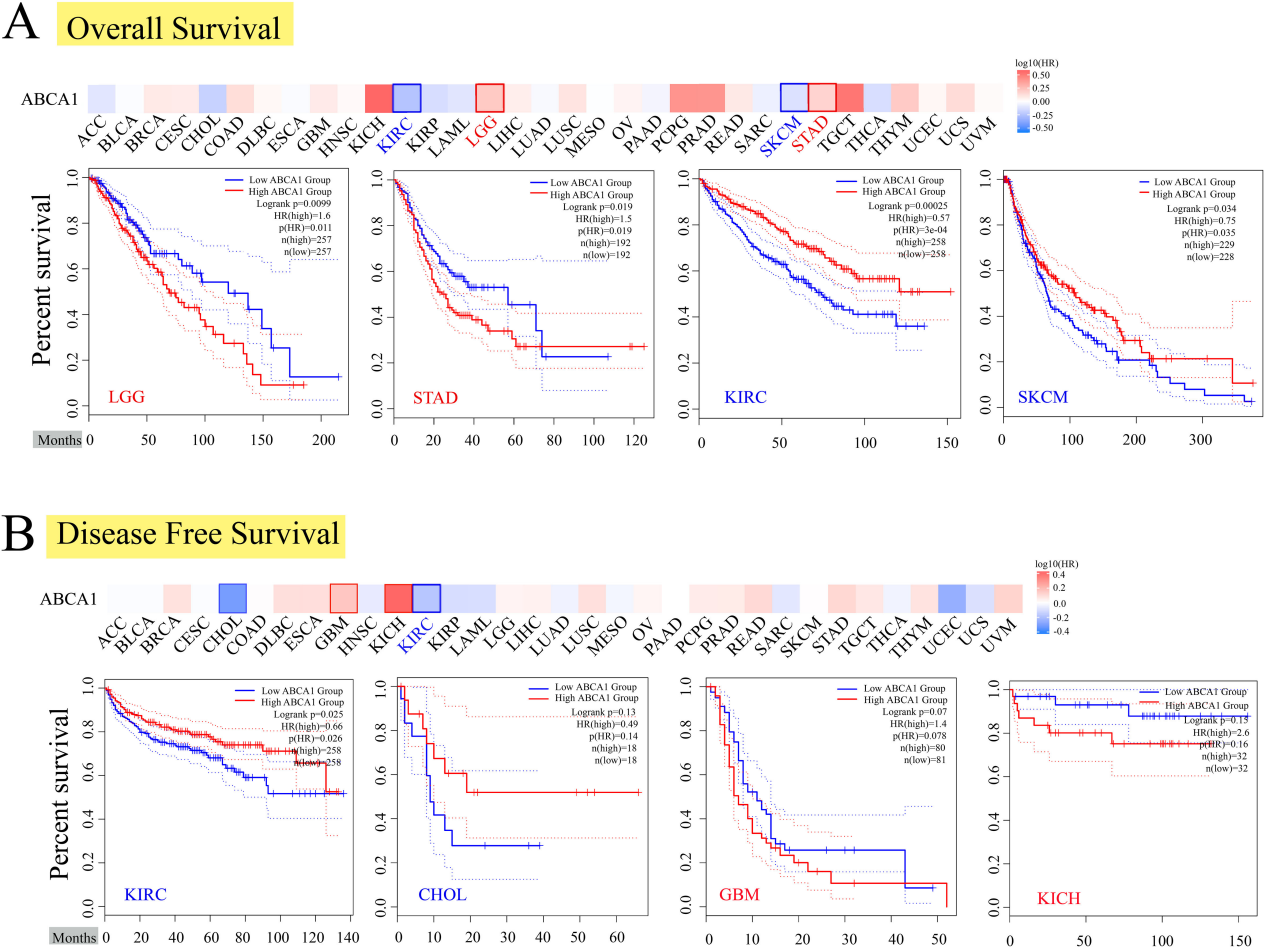
Correlation between *ABCA1* gene expression and survival prognosis of cancers in TCGA. We used the GEPIA2 tool to perform overall survival **(A)** and disease-free survival **(B)** analyses of different tumors in TCGA by *ABCA1* gene expression. The survival map and Kaplan-Meier curves with positive results are given. Red label indicated positively correlated, blue label indicated negatively correlated.

To further investigate the relationship between ABCA1 expression and the prognosis of different cancer patients, we used the expression level of ABCA1 to classify cancer patients into high- and low-expression groups, in which the TCGA and GEO datasets were used to explore the correlation between the expression level of ABCA1 and the prognosis of different tumour patients. As shown in **Figure 2A**, high expression of ABCA1 was associated with poor overall OS in LGG (*P* = 0.0099) and STAD (*P* = 0.019) patients, whereas low expression of the ABCA1 gene was associated with poor OS in KIRC (*P* = 0.00025) and SKCM (*P* = 0.034) patients. The disease-free survival (DFS) data in **Figure 2B** show that, in the TCGA cohort, poor DFS in KIRC patients (*P* = 0.025) and CHOL patients (*P* = 0.13) was associated with reduced ABCA1 expression, whereas high ABCA1 expression was associated with poor DFS in GBM patients (*P* = 0.07) and KICH patients (*P* = 0.15).

### 3.3 Characteristics of *ABCA1* mutations in the TCGA pan-cancer cohort

**Figure 3:**
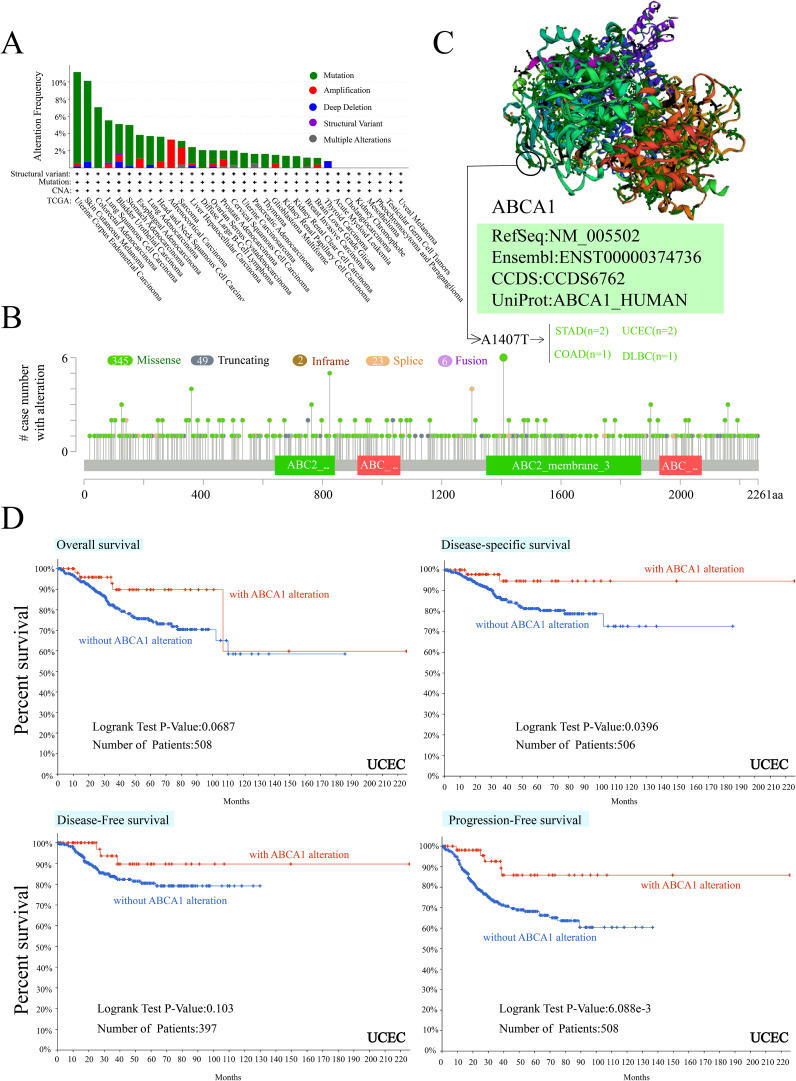
Mutation feature of *ABCA1* in different tumors of TCGA. We analyzed the mutation features of *ABCA1* for the TCGA tumors using the cBioPortal tool. The alteration frequency with mutation type **(A)** and mutation site **(B)** are displayed. We display the mutation site with the highest alteration frequency (A1407T) in the 3D structure of *ABCA1* **(C)**. We also analyzed the potential correlation between mutation status and overall, disease-specific, disease-free and progression-free survival of UCEC **(D)** using the cBioPortal tool.

We observed *ABCA1* gene changes in different tumour samples in the TCGA cohort. As shown in **Figure 3A**, patients with UCEC tumours with “mutation” as the main type had the highest frequency of *ABCA1* gene changes (> 10%). In ACC patients, the “amplified” type of CNA was the dominant type, with a frequency of change greater than 3%. Notably, all patients with genetically altered ACC had *ABCA1* amplification, whereas all patients with genetically altered COAD, diffuse large B-cell lymphoma, uterine sarcoma, THYM, KIRP, and KIRC had *ABCA1* mutations. **Figure 3B** further shows the types, loci and number of cases of *ABCA1* gene alterations. We found that missense mutations were the main type of *ABCA1* gene alteration, and the A1407T mutation of the ABC2_membrane_3 domain detected in 2 UCEC patients, 2 STAD patients, 1 COAD patient, and 1 DLBC patient could induce missense mutation of the *ABCA1* gene. **Figure 3C** clearly shows the three-dimensional structure of the ABCA1 protein and the A1407T residue from the front. In addition, we analysed the potential associations between genetic alterations in *ABCA1* and clinical survival outcomes in different types of cancers. As shown in **Figure 3D**, in terms of overall survival (*P* = 0.0687), there was no significant difference in prognosis between most UCEC patients with *ABCA1* changes and UCEC patients without *ABCA1* changes. The prognosis of UCEC patients with *ABCA1* changes was worse than that of UCEC patients without *ABCA1* changes around the 100th month. These results suggest that *ABCA1* mutations may have little effect on overall survival. In terms of disease-specific survival (*P* = 0.0396), the survival rate in the group with *ABCA1* gene alterations was significantly greater than that in the group without alterations, suggesting that *ABCA1* mutations may affect disease-related mortality. In terms of disease-free survival (*P* = 0.103), the difference between patients with and without ABCA1 mutations was not significant, suggesting that *ABCA1* gene mutations may have a limited effect on the risk of recurrence. In terms of progression-free survival (*P* = 6.088e-3), the prognosis of UCEC patients with *ABCA1* alterations were generally better than that of UCEC patients without *ABCA1* alterations, suggesting that *ABCA1* mutations may affect the risk of disease progression. The chart clearly shows that *ABCA1* gene alterations may have different effects on different survival measures, suggesting that it may play a role in tumour progression and the survival rate of specific cancers.

### 3.4 Genetic immunohistochemical analysis data revealed the expression of *ABCA1* at the protein level

**Figure 4:**
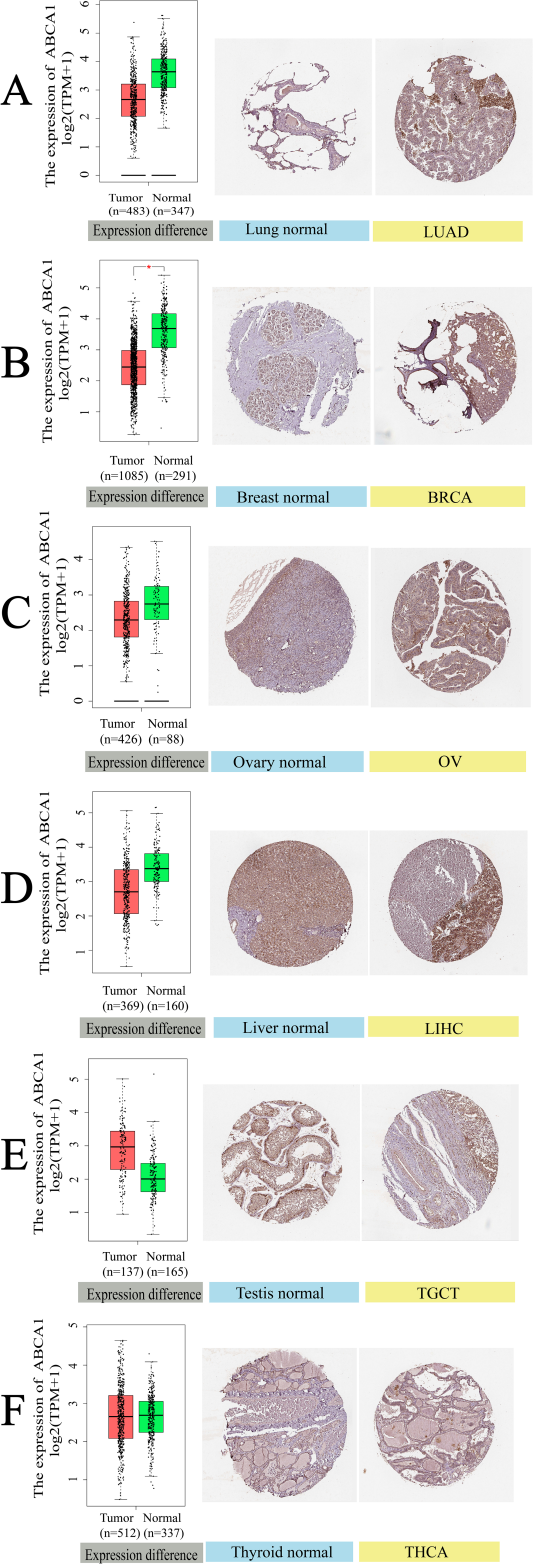
Gene expression and immunohistochemistry of *ABCA1* in tumors and normal tissues. For the type of LUAD, BRCA, OV, LIHC, TGCT, and THCA in the TCGA project, the corresponding normal tissues of the GTEx database were included as controls. The box plot data were supplied. *P < 0.05. We also used the HPA database to obtain immunohistochemical results of *ABCA1* in tumors and normal tissues. The protein expression of GPC2 in immunohistochemical images of tumor (left) and normal (right) groups. **(A)** Lung. **(B)** Breast. **(C)** Ovary. **(D)** Liver. **(E)** Testis. **(F)** Thyroid.

**Figure 4** illustrates the differential expression of ABCA1 in normal and tumour tissues across six cancer types, as analysed via IHC. The results revealed the downregulation of ABCA1 in LUAD, BRCA, OV, and LIHC tumour tissues compared with the corresponding normal tissues. In contrast, *ABCA1* expression was upregulated in TGCT and THCA. Moreover, the IHC analysis further clarified these differences. First, normal lung tissue contained many erythrocytes and alveolar capillaries. In contrast, LUAD cells had loose chromatin and vesicular nuclei. Second, normal mammary cells were uniform in size and shape with some amount of cytoplasm and visible vacuoles. In rare cases, tumour cells may grow significantly to form solid nests or nodules, whereas BRCA cells may form tubular, fibroadenomatous, or acinar forms. Third, normal ovarian cells have a single nest, sheet, or papillary layout, whereas ovarian cancer cells have a sieve-like, microcystic layout; thus, they are sieve-like microcystic structures. Fourth, normal hepatocytes contain abundant ectochromatin, and some of them have binucleated or polyploid nuclei. Some LIHC cells contain lipid vacuoles and the cellular changes in their malignant tumours are usually minor. Fifth, most testicular cells have cuboidal epithelium and slits. In contrast, fibrovascular septa are observed in TGCT cells, separating the cells into tightly packed clusters and nests of cells, and aggregation of lymphocytes along the septa can be observed. Sixth, in normal thyroid cells, a few follicular cells are large and polygonal with well-defined borders and large nuclei. Tightly clustered follicles and papillae are symptoms of THCA, and some of THCA tumour cells are invasive. These IHC findings highlight the heterogeneous expression patterns of ABCA1 across different cancers, suggesting its potential role in tumour progression and malignancy-specific regulatory mechanisms; for example, ABCA1 is involved in cholesterol efflux, and differences in ABCA1 expression can affect cholesterol metabolism and HDL biosynthesis; provide nutrients and signalling molecules required for tumour cells to grow; and subsequently affect tumour cell proliferation, invasion and metastasis. ABCA1 may also affect immune cell function by regulating cholesterol levels in the tumour microenvironment, helping tumours evade immune surveillance.

### 3.5 Immunoinfiltration analysis of *ABCA1* might explain its influence on the prognosis and survival of cancer patients

**Figure 5:**
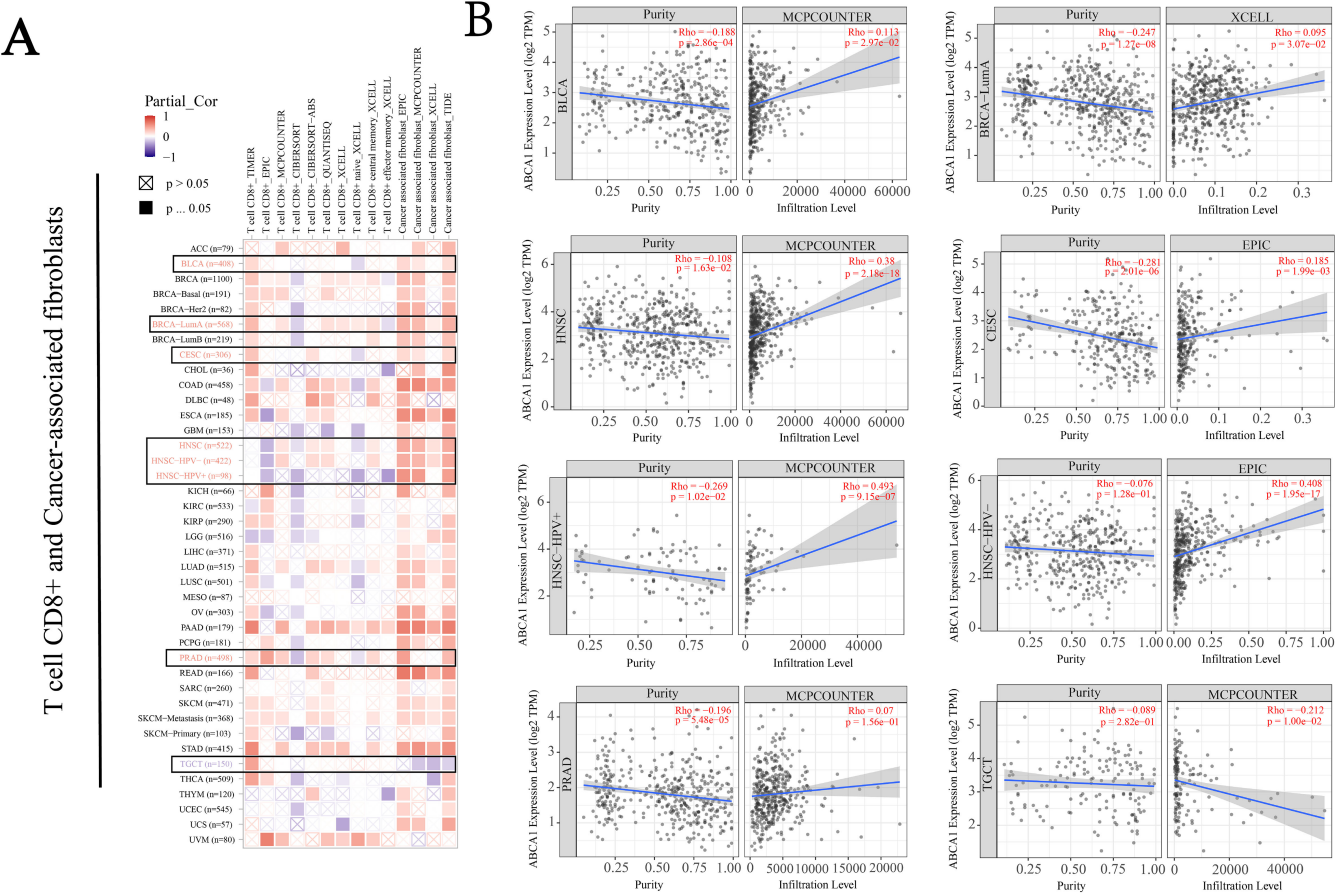
Correlation analysis between *ABCA1* expression and immune infiltration of cancer-associated fibroblasts. We used different algorithms (EPIC, MCPCOUNTER, and XCELL, among others) to explore potential correlations between ABCA1 gene expression levels and levels of infiltration of all types of CD8+T cells and cancer-associated fibroblasts in TCGA. **(A)** Heat maps of ABCA1’s association with infiltration of CD8+T cells and tumor-associated fibroblasts. **(B)** This plot is a representative scatter plot between ABCA1 and tumor-associated fibroblast infiltration.

According to previous findings, cancer-associated fibroblasts in the stroma are involved in the regulation of immune cells infiltrating different tumours (18). As shown in **Figure 5A**, we used the EPIC and MCPCOUNTER algorithms to study the correlation between the infiltration of CD8+ T cells and cancer-related fibroblasts and the expression of *ABCA1* in different malignant tumours. For example, according to the TIMER algorithm, CD8+ T-cell infiltration in BLCA, BRCA-LumA, CESC, PRAD and TGCT cancers was positively correlated with *ABCA1* expression, whereas according to the CIBERSORT algorithm T-cell infiltration in all cancers was negatively correlated or not correlated with *ABCA1* expression. The estimated filtration values of cancer-associated fibroblasts from BLCA, CESC, BRCA-LumA, PRAD, HNSC, and HNSC (human papillomavirus +/-) tumour samples were statistically positive for *ABCA1* expression. However, we observed a negative correlation between cancer-associated fibroblast infiltration in TGCT and *ABCA1* expression. **Figure 5B** shows the scatterplot data of the above cancer-associated fibroblast infiltration generated by the algorithm. The level of *ABCA1* expression in TGCT according to the MCPCOUNTER algorithm (Rho = -0.212, *P* = 1.00e - 02) was inversely correlated with the amount of cancer-associated fibroblast infiltration. In contrast, *ABCA1* expression levels based on the MCPCOUNTER algorithm were higher in BLCA (Rho = 0.113, *P* = 2.97e - 02), HNSC (Rho = 0.38, *P* = 2.18e - 18), HNSC-HPV+ (Rho = 0.493, *P* = 9.15e - 07), PRAD (Rho = 0.07, *P* = 1.56e - 01) and BRCA-LumA (Rho = 0.095, *P* = 3.07e - 02) with the XCELL algorithm, as was the number of cancer-associated fibroblast infiltrates with the EPIC algorithm in CESC (Rho = 0.185, *P* = 1.99e - 03) and HNSC-HPV- (Rho = 0.408, *P* = 1.95e - 17). Infiltration was positively correlated with ABAC1 expression.

**Figure 6:**
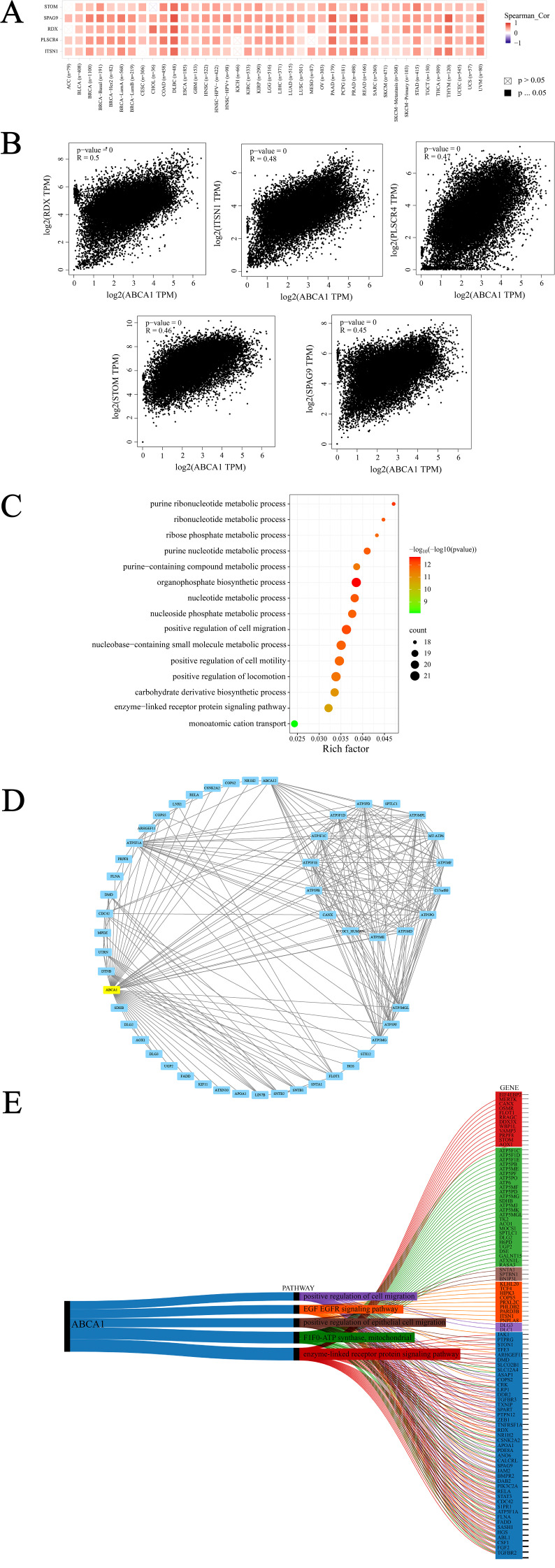
*ABCA1*-related gene enrichment analysis. **(A)** The corresponding heatmap data in the detailed cancer types are displayed. **(B)** Using the GEPIA2 approach, we also obtained the top 100 *ABCA1*-correlated genes in TCGA projects and analyzed the expression correlation between *ABCA1* and selected targeting genes, including RDX, ITSN1, PLSCR4,STOM and SPAG9. **(C)** Based on the *ABCA1*-binding and interacted genes, KEGG pathway analysis was performed. **(D)** We first obtained the available experimentally determined *ABCA1*-binding proteins using the STRING tool. **(E)** Sankey diagram of *ABCA1*: The picture demonstrates the pan-oncogene and pathway associated with *ABCA1* gene. (*ABCA1* single gene is highly associated with other pan-cancer pathways are enzyme-linked receptor protein signaling pathway, F1F0-ATP synthase, mitochondria, positive regulation of epithelial cell migration, EGF epidermal growth factor receptor signaling pathway, and positive regulation of cell migration).

### 3.6 Enrichment analysis suggests that the “organophosphate biosynthetic process” might be involved in the effect of ABCA1 on tumour pathogenesis

First, we used the GEPIA2 tool to integrate all tumor expression data of TCGA to generate the top 100 targeted genes related to *ABCA1* expression, and selected the five genes with the strongest correlation according to Pearson correlation coefficient index. They are RDX (Radixin), ITSN1 (Intersectin 1), PLSCR4 (Phospholipid Scramblase 4), STOM (Stomatin) and SPAG9 (Sperm associated antigen) 9), these genes play roles in plasma membrane junction, cytoskeletal recombination, lipid distribution and cell signaling, membrane transport and ion channels, and cell proliferation, respectively. As shown in **Figure 6A**, we used the TIMER tool to combine relevant data and found that the expression of *ABCA1* was strongly positively correlated to these five genes in most cancer types. Then, GEPIA2 tool was used again for correlation analysis and the dot plot was generated, as shown in **Figure 6B**, ABCA1 expression was positively correlated with radixin (RDX) (R = 0.5), intersectin 1 (ITSN1) (R = 0.48), phospholipid scramblase 4 (PLSCR4) (R = 0.47), stomatin (STOM) (R = 0.46) and sperm-associated antigen 9 (SPAG9) (R = 0.45) (*P* < 0.001). To better explore the molecular mechanism of carcinogenesis involving the ABCA1 gene, we screened genes that target ABCA1-binding proteins and genes related to ABCA1 expression and conducted a series of pathway enrichment analyses. The bubble map obtained is shown in **Figure 6C**. The data showed that the “organophosphate biosynthetic process” may be closely related to the effect of ABCA1 on tumour pathogenesis. Moreover, we used the STRING tool to identify 50 ABCA1-binding proteins supported by experimental evidence, and the interaction network of these proteins is shown in **Figure 6D**. Moreover, as shown in **Figure 6E**, the top 10 genes highly correlated with the ABCA1 single gene and other panoncogenes were TGFBR2, FGF2, CSF1, ABL1, HGS, SASH1, FADD, FLNA, ATP5F1A, and S1PR1. The five major pathways in which the ABCA1 single gene was highly correlated with other pan-cancer pathways included the enzyme-linked receptor protein signalling pathway, F1F0-ATP synthase, mitochondria, positive regulation of epithelial cell migration, the EGF epidermal growth factor receptor signalling pathway, and positive regulation of cell migration.

### 3.7 The expression of ABCA1 may be regulated by miRNAs

**Figure 7:**
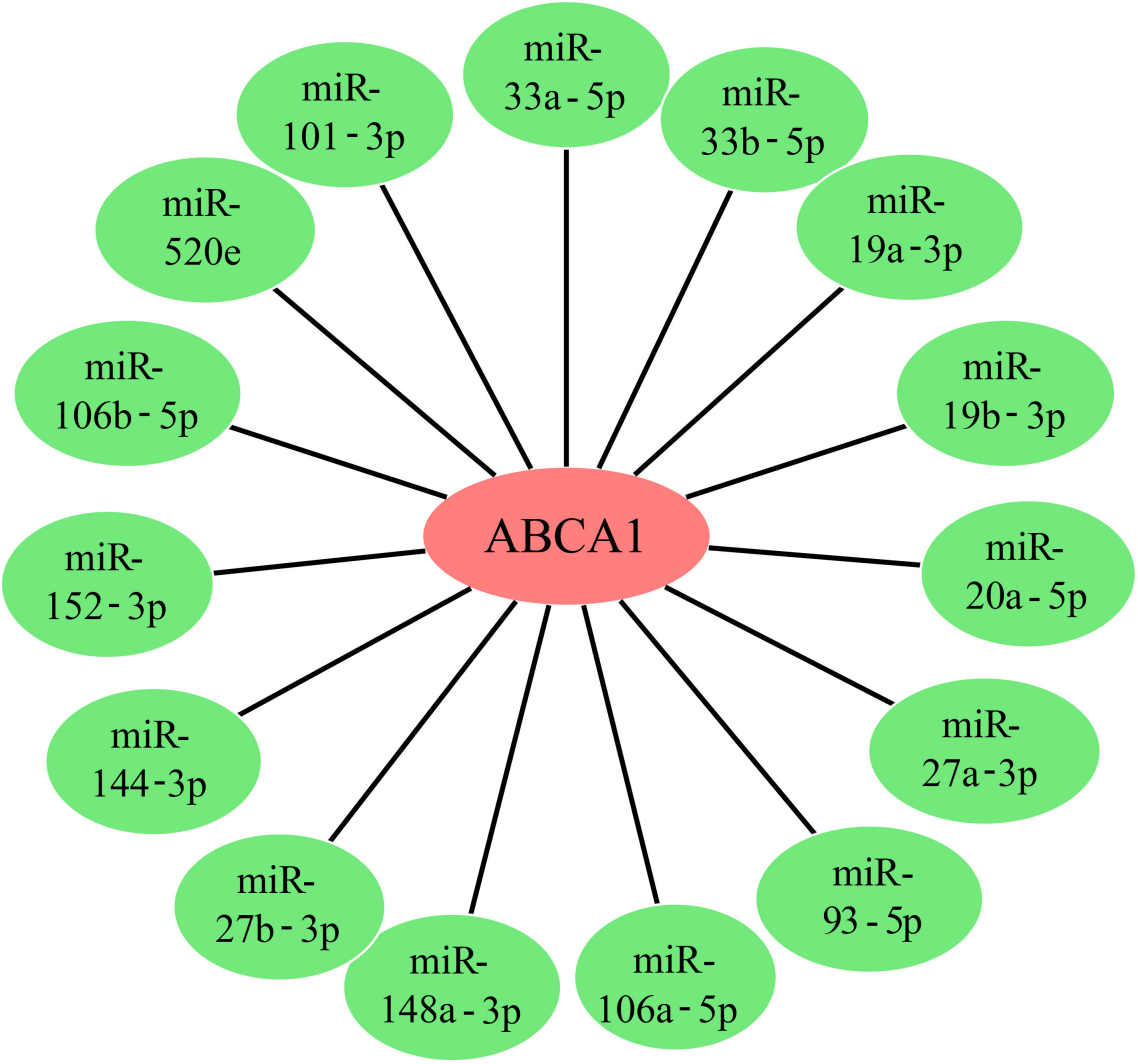
*ABCA1* gene upstream miRNA prediction. We predicted miRNAs for the target gene *ABCA1* using the starbase database, and 15 target miRNAs that can represent the target gene *ABCA1* are given in the figure.

A class of noncoding RNAs called microRNAs (miRNAs) have emerged as key regulators of gene expression, primarily acting at the posttranscriptional level. To determine whether the expression of ABCA1 is regulated by some miRNAs, we obtained upstream miRNAs that may bind to ABCA1 through the starBase database and a total of 15 representative target miRNAs were identified according to the number of predicted databases, as shown in **Figure 7**. These miRNAs were miR-33a-5p, miR-33b-5p, miR-19a-3p, miR-19b-3p, miR-20a-5p, miR-27a-3p, miR-106a-5p, miR-148a-3p, miR-27b-3p, miR-144-3p, miR-152-3p, miR-106b-5p, miR-520e, miR-93-5p, and miR-101-3p. Florian Peters et al. described the regulatory effects of miR-33 and miR-34a on ABCA1 in ageing eyes (19). Joel Vega-Badillo et al. reported that the relative expression of miR-33a and miR-144 was negatively correlated with the level of ABCA1 protein (20). Da-Peng Bi et al. also reported that miR-183 promoted proliferation and inhibited apoptosis by degrading ABCA1 in colon cancer (21). From these perspectives, the expression of ABCA1 may be regulated by multiple miRNAs, leading to a role in cancer development.

## 4 Discussion

ABCA1 is an ATP-binding cassette (*ABC*) subfamily A export source, and the cryo-electron microscopy structure of ABCA1 in humans has a nominal resolution of 4.1 Å for the overall structure and 3.9 Å for the large extracellular structural domain (22). It regulates cholesterol and phospholipid contents in the plasma membrane and affects lipid rafts, microparticle (MP) formation, and cellular signalling, and impaired ABCA1 function and altered cholesterol homeostasis may affect many different organs and be involved in the pathophysiology of a wide range of diseases (2). Therefore, we conducted a comprehensive study of the *ABCA1* gene in different tumours on the basis of data from the TCGA, CPTAC, and GEO databases, as well as molecular characterization of gene expression, gene alterations, and protein phosphorylation.

In this study, ABCA1 was highly expressed in most tumour tissues compared with normal tissues. However, different conclusions can be reached in different tumours. Immunohistochemical analysis of the *ABCA1* gene revealed that ABCA1 expression in THCA tissues was upregulated compared with that in corresponding normal tissues. In line with these findings, Ji-Hye Park et al. reported that ABCA1-mediated EMT promoted thyroid carcinoma malignancy through the ERK/Fra-1/ZEB1 pathway (11). However, there are few reports on the role of ABCA1 in THCA tumours. These results may provide a new breakthrough point for exploring the relationship between ABCA1 and THCA.

In glioblastoma, we found that low ABCA1 expression in GBM patients was associated with poor DFS (*P* = 0.07), but the expression level of ABCA1 in GBM tumour tissues was greater than that in corresponding normal tissues (*P* < 0.05). In line with this conclusion, Deven Patel et al. reported that LXRβ controls glioblastoma cell growth, lipid balance and immune regulation independently of ABCA1 and that high cell density induces ABCA1 expression in glioblastoma cells (23). It is worth mentioning that we found that the anticancer drug temozolomide (TMZ) is often used in GBM treatment, whereas Shao-Ming Wang et al. revealed that the expression level of the *ABCA1* gene is an indicator of TMZ treatment efficiency. ABCA1 inhibition has been proposed as a potential strategy for the treatment of TMZ-resistant GBM (24). Moreover, Shiqun Wang et al. noted that with the progression of tumours, the expression level of ABCA1 in TAMs is positively correlated with the TAM (tumor associated macrophage) population in GBM tumour tissue. It was concluded that downregulating ABCA1 levels could promote TAM polarization to an inflammatory phenotype and control GBM tumour growth (25). Therefore, the expression level of ABCA1 has a certain degree of influence on the development, treatment and prognosis of GBM tumours; however, further study of the role of ABCA1 in the treatment of GBM tumours is still needed.

In cutaneous melanoma, we found a correlation between low ABCA1 expression and poor OS prognosis in SKCM patients (*P* = 0.034), and although the correlation was not high, this finding does not indicate that ABCA1 has low or even now association with the development of this tumour. Ambroise Wu et al. reported that high levels of ABCA1 transporter proteins in human melanoma are associated with poor prognosis and that depletion or inhibition of ABCA1 activity affects the invasive capacity of aggressive melanoma cells and regulates the lateral organization of the plasma membrane of melanoma cells by increasing cholesterol levels and preventing the formation of active adherent plaques, thus suggesting that ABCA1 is a potential metastatic marker for melanoma (26). Guillaume Hanouna et al. investigated the role of extracellular calpain in a melanoma model by blocking extracellular activity or externalization and ultimately reported that decreasing ABCA1 activity in probenecid could prevent calpain externalization to limit melanoma angiogenesis and progression (27). Hanwen Wang et al. reported that ABCA1 is a downstream target of ALKBH5-mediated m^6^ demethylation, and data showing the functional value of the key demethylase ALKBH5-mediated m^6^ modification of SKCM progression suggest that the ALKBH5-m^6^A-ABCA1 axis serves as a potential therapeutic target in SKCM (28). In conclusion, more evidence in addition to the above studies is needed concerning the potential role of ABCA1 in the development of cutaneous melanoma.

According to our analysis, high expression of ABCA1 was associated with poor OS in patients with STAD (*P* = 0.019). Wei Tang et al. developed a 5-CMG prognostic signature (including ABCA1) that can effectively predict the prognosis of patients with gastric cancer and their response to chemotherapy plus PD-1 inhibitors (29). In addition, Ran Wang et al. reported that after LXR-β was activated with GW3965, ABCA1 was significantly upregulated, and the proliferation of gastric cancer cells was significantly inhibited (30). Xing Wang et al. also reported that PILRB can reduce the cholesterol level of STAD cells by altering the expression levels of ABCA1 and SCARB1 (31). Qianhui Liu et al. reported that PUF60 enhances the chemotherapeutic resistance of STAD cells by actively eliminating chemotherapeutic agents through recombinant ATP-binding cassette transporter A1 (ABCA1) and ATP-binding cassette subfamily C member 1 (ABCC1) (32). These findings suggest that ABCA1 plays a role in the proliferation and drug resistance of STAD, and if further studies are carried out, ABCA1 may also be found to be a key factor in inhibiting the spread of gastric cancer cells.

By analysing the results, we found a correlation between low expression of ABCA1 and poor OS (*P* = 0.00025) and DFS (*P* = 0.025) in renal clear cell carcinoma patients. A study by Chan-Juan Zhang et al. revealed that the anticancer effect of celastrol in renal clear cell carcinoma is caused by the activation of LXRα signalling to trigger autophagy and LD degradation, which then promotes ABCA1-mediated cholesterol efflux and impairs EMT progression. This ultimately inhibits renal clear cell proliferation, migration, and invasion, as well as tumour growth (33). Zhijuan Liang et al. reported that CYP27A1 regulates cholesterol homeostasis by activating LXRs/ABCA1 and plays a regulatory role in the proliferation and migration of renal clear cell carcinoma (34). Fabiana Perrone et al. suggested that cholesterol efflux mediated by serum transporters (including ABCA1) and passive diffusion affect the clinical outcome of ICI treatment in patients with advanced renal cell carcinoma (35). In addition, ABCA1 and ABCB1 are both transporters. Bin Huang et al. also reported that PKCϵ inhibits the isolation and dryness of lateral population cells by inhibiting the ABCB1 transporter and PI3K/Akt and MAPK/ERK signalling in the renal cell line 769P (36), suggesting that ABCA1 may also play an inhibitory role in the development of renal cell cancer cells. There are few data on the effect of ABCA1 on renal clear cell carcinoma. However, most of the current data show that ABCA1 is related to the proliferation, invasion and metastasis of cancer, but whether ABCA1 affects the prognosis of cancer patients by affecting the development of renal clear cell cancer cells needs further exploration.

In addition, we inferred that the expression of ABCA1 is associated with the development of breast cancer. Hailing Pan et al. reported that the expression of ABCA1 in TNBC tissues is greater than that in noncancerous breast tissues. The high expression of ABCA1 in TNBC tissues was significantly correlated with histological grade, and the results indicated that ABCA1 is a specific marker for TNBC (37). Dhiya Salih Al-Shumary et al. reported that the high expression of ABCA1 and ABCA3 in AMJ13 and MCF-7 breast cancer cell lines is associated with drug resistance and plays an important role in drug resistance (38). S Schimanski et al. reported strong expression of ABCA3 and ABCA1 in the endothelial layer of the normal breast epithelium. Strong expression of ABCA1 was associated with positive lymph nodes, but there was no significant correlation with tumour recurrence or breast cancer-specific survival (39). Mai O Kadry et al. reported that metabolomic integrated genomics analysis may hold promise for understanding multidrug resistance phenotypes in MCF-7 breast cancer cells exposed to adriamycin by modulating the ABCA1/EGFR/P53/PI3k/PTEN signalling pathway (40). In addition, Yuan Yuan et al. reported that ATP-binding box (ABC) transporters play a role in evaluating prognosis, predicting immunity and guiding treatment in patients with breast cancer (41). Therefore, the role of ABCA1 in breast cancer is widely understood, including prediction, drug resistance, treatment and prognosis, which provides a promising direction for further research and potential BRCA therapeutic intervention.

Through the above discussion, we summarized the possible predictive, controlling and therapeutic effects of ABCA1 in cancer. These findings are of great clinical importance, but our research method still has certain limitations. On the one hand, we relied mainly on the analysis of health data and literature references, not experimental verification; on the other hand, our research depth is insufficient. However, in this study, further clarification of the biological mechanism was difficult. Despite these limitations, which do not reduce the reliability of our conclusions, our findings still have reference value for further explorations of the practical application of ABCA1 in cancer treatment and will provide a more effective and accurate means for the clinical treatment of cancer tumours in the future.

## 5 Conclusions

In summary, our first pan-cancer analysis of ABCA1 revealed a statistical correlation between ABCA1 expression and clinical prognosis, genetic variation, immune cell infiltration in different types of tumours and that ABCA1 is aberrantly expressed in various cancers, which further demonstrates the important role of ABCA1 in tumorigenesis and tumour immunity in a variety of cancers. Therefore, our comprehensive pan-cancer analysis helps elucidate the involvement of ABCA1 in tumorigenesis, development, treatment, and prognosis from multiple perspectives, reflecting the great potential of ABCA1 as a biomarker for predicting the immunotherapeutic response and a promising therapeutic target.

## Glossary

ABCA1: ATP Binding Cassette Transporter A1
ABCC1: ATP Binding Cassette Subfamily C Member 1
ABCB1: ATP Binding Cassette Subfamily B Member 1
ABL1: ABL proto-oncogene 1(non-receptor tyrosine kinase)
ATP5F1A: ATP Synthase F1 Subunit Alpha
ABCA3: ATP Binding Cassette Subfamily A Member 3
apoA-I: apolipoprotein A-I
ACC: adenoid cystic carcinoma
BRCA: breast invasive carcinoma
BLCA: urothelial carcinoma of the bladder
CESC: cervical squamous carcinoma
CYP27A1: Cytochrome P450 27A1
CHOL: cholangiocarcinoma
COAD: colorectal adenocarcinoma
CNA: copy number changes
CPTAC: Clinical Proteomic Tumor Analysis Consortium
CSF1: Colony Stimulating Factor 1
DFS: disease-free survival
DLBC: diffuse large B-cell lymphoma
ESCA: esophageal carcinoma
EMT: Epithelial–mesenchymal transition
FADD: Fas-associating protein with a novel death domain
FLNA: Filamin A
FGF2: Fibroblast growth factor 2
GEO: Gene Expression Omnibus
GBM: glioblastoma multiforme
HNSC: head and neck squamous cell carcinoma
HPA: Human Protein Atlas
HGS: Hepatocyte Growth Factor-Regulated Tyrosine Kinase Substrate
ITSN1: Intersectin 1
KEGG: Kyoto Encyclopedia of Genes and Genomes
KIRC: clear cell carcinoma of kidney
KIRP: papillary cell carcinoma of kidney
KICH: phaeochromocytoma of kidney
LGG: low-grade glioma
LUSC: squamous cell carcinoma of the lung
LIHC: liver hepatocellular carcinoma
LUAD: lung adenocarcinoma
LAML: Acute Myeloid Leukemia
nHDL: newborn high density lipoprotein
NNMT: Nicotinamide N-methyl transferase
OS: overall survival
OV: ovarian plasma cystic adenocarcinoma
PILRB: Paired Immunoglobin Like Type 2 Receptor Beta
PUF60: poly-U binding sp-licing factor 60 Kda
PRAD: Prostate adenocarcinoma
PCPG: pheochromocytoma and paraganglioma
PAAD: Pancreatic adenocarcinoma
PLSCR4: Phospholipid Scramblase 4
READ: rectal adenocarcinoma
RASSF1: Ras Association Domain Family Member 1
RDX: Radixin
SASH1: SAM And SH3 Domain Containing 1
S1PR1: Sphingosine-1-Phosphate Receptor 1
SCARB1: scavenger receptor class B type I
STAD: gastric adenocarcinoma
SKCM: Skin Cutaneous Melanoma
STOM: Stomatin
SPAG9: Sperm-associated antigen 9
TGFBR2: Transforming growth factor beta receptor 2
TAM: tumor associated macrophage
TMZ: Temozolomide
TNBC: Triple-negative breast cancer
TCGA: The Cancer Genome Atlas
TGCT: testicular germ cell tumor
THCA: thyroid cancer
THYM: thymic carcinoma
UCEC: Uterine Corpus Endometrial Carcinoma
